# Improvement in the Between-Class Variance Based on Lognormal Distribution for Accurate Image Segmentation

**DOI:** 10.3390/e24091204

**Published:** 2022-08-29

**Authors:** Walaa Ali H. Jumiawi, Ali El-Zaart

**Affiliations:** Department of Mathematics and Computer Science, Faculty of Science, Beirut Arab University, Beirut 11072809, Lebanon

**Keywords:** between-class variance, thresholding, images segmentation, lognormal distribution, Otsu’s method, right-skewed distribution

## Abstract

There are various distributions of image histograms where regions form symmetrically or asymmetrically based on the frequency of the intensity levels inside the image. In pure image processing, the process of optimal thresholding tends to accurately separate each region in the image histogram to obtain the segmented image. Otsu’s method is the most used technique in image segmentation. Otsu algorithm performs automatic image thresholding and returns the optimal threshold by maximizing between-class variance using the sum of Gaussian distribution for the intensity level in the histogram. There are various types of images where an intensity level has right-skewed histograms and does not fit with the between-class variance of the original Otsu algorithm. In this paper, we proposed an improvement of the between-class variance based on lognormal distribution, using the mean and the variance of the lognormal. The proposed model aims to handle the drawbacks of asymmetric distribution, especially for images with right-skewed intensity levels. Several images were tested for segmentation in the proposed model in parallel with the original Otsu method and the relevant work, including simulated images and Medical Resonance Imaging (MRI) of brain tumors. Two types of evaluation measures were used in this work based on unsupervised and supervised metrics. The proposed model showed superior results, and the segmented images indicated better threshold estimation against the original Otsu method and the related improvement.

## 1. Introduction

Thresholding-based segmentation is the most commonly used technique for segmenting images. Thresholding is a simple and effective process to find an adequate value of gray level for separating objects from their background [1]. Various techniques were proposed in the literature in this regard. In an ideal case, the gray level histogram has the symmetric distribution of two regions representing foregrounds and backgrounds, respectively, such that the threshold can be chosen at the bottom between the two regions [2]. Image segmentation is a sensitive and difficult process in computer vision and image analysis applications, where no segmentation algorithm can give the best result for any type of image [3]. Segmentation techniques tend to make understandable images after grouping objects and re-represent the image in separated segments. In general, the segmentation techniques rely upon two cases of intensity level presence in an image: discontinuity and similarity. In the first case, the segmentation technique relies on the sudden changes in intensity level; this represents the edges of intensities inside images, while in the second case, the technique relies on the similar presence of intensity levels that represent one area based on predefined criteria [4].

There are different types of images where intensity levels have different forms of distribution. Images with right-skewed histograms exist in every field, and sometimes images affected by specific lighting conditions generate the skewness of right histograms in images. This condition can be implemented as simulated images in this regard, as we did in this work to test the proposed model. Medical Resonance Imaging (MRI) is one of the most effective technologies that produce anatomical images [5]. Some MRI brain tumor images have specific features that make the segmentation process difficult such as heterogeneous intensities around the tumor, background noises, fuzzy boundaries, and less contrast between brain tissues [6,7]. The proposed model can play an important role in determining the optimal threshold for these cases. Accurate segmentation provides reliable object detection in medical diagnosis, observing the segmented shape inside the given images [8,9], and this is every imaging specialist and radiologist’s goal.

The original Otsu’s method is one of the thresholding techniques that uses Gaussian distribution in its objective function [4,10], based on the assumption that an image histogram has symmetric modes. In the case where the image histogram has nonsymmetric modes (skewed to the right), the original Otsu method showed some problems in the image thresholding result. In [3], the Otsu method was improved based on its original formula but using only the mean value of lognormal distribution on its objective function. However, theoretically, this improvement has some problems because the formula of the original Otsu is based on the Gaussian, and in [3], the authors used the mean of lognormal instead of the mean of Gaussian. Our proposed method uses the definition of lognormal distribution in the development of the objective function to obtain an improved formula of the original Otsu method. The proposed model has been evaluated and compared with the original Otsu method and another relevant method presented in the literature. The main contributions of this research are:

- Developing a formula of the between-class variance based on lognormal distribution;
- Introducing an accurate segmentation model for images that have a right-skewed histogram distribution and handling the challenges of finding the optimal threshold in such types of images;
- Implementing a boosted and inclusive segmentation algorithm that measures segmentation results in parallel using supervised and unsupervised evaluation.

This research is divided as follows: The related work is stated in Section 2, with a brief introduction to the Otsu method and lognormal distribution. In Section 3 we explained the proposed between-class variance. The performance evaluations are stated in Section 4. The results are discussed in Section 5. Finally, the conclusion and future work are stated in Section 6.

## 2. Related Work

Image segmentation is one of the major topics for image processing researchers. Various methods have been presented in the literature. Each method has a different development approach for defining good segmentation based on the optimal threshold. Although thresholding is the simplest process in image segmentation, the optimal threshold that accurately separates objects from the background remains a difficult task [10]. Gray-level histogram-based thresholding is the most used method in image segmentation. The easiest task when a given image f (x, y) has dark objects on a bright background or vice versa, representing a bimodal histogram, is to separate this histogram using the threshold value that can be located in the valley of the histogram classes. According to the thresholding definition, the segmented image g(x, y) is a binary image composed using pixels comparison from the image f(x, y) with the optimal threshold T [4]. One of the most used thresholding methods in this form is Otsu’s method.

### 2.1. The Original Otsu’s Method 

Otsu’s method relies on the variance of the gray level to obtain the threshold value that groups the image pixels into a binary form, the object, and the background. Otsu algorithm uses the definition of Gaussian distribution. In general, the threshold value is selected to maximize the between-class variance. The Otsu algorithm for finding the global threshold is categorized in the following [4,10].

#### 2.1.1. The Probability and Gray Mean Value

The intensity level of the image *f(x, y)* is [0, 1, 2, … L − 1]. The threshold value *t* (0 ≤ *t* ≤ L − 1) split *f(x, y)* into two categories: object and background. The probability of object class is $P_{o}$, and the probability of background class is $P_{b}$, as defined in Equations (1) and (2).

(1) $$P_{o}=\overset{t}{\underset{i=0}{\sum }}\;P_{i}\;$$

(2) $$P_{b}=\overset{L-1}{\underset{i=t+1}{\sum }}\;P_{i}\;$$

where $P_{i}$ is the ratio of $n_{i}$, and $n_{i}$ is the number of pixels in the intensity level *i* of the entire image, indicating the probability of intensity level *i*.

(3) $$P_{i}=\frac{n_{i}}{n\;}\;$$

The values ${µ}_{o}$ and ${µ}_{b}$ represent the mean of object and background, respectively, as defined in Equations (4) and (5).

(4) $$\mu_{o}^{}\left(t\right)=\overset{t}{\underset{i=0}{\sum }}\;\;\frac{iP_{i}}{P_{o}}\;$$

(5) $$\mu_{b}^{}\left(t\right)=\overset{L-1}{\underset{i=t+1}{\sum }}\;\;\frac{iP_{i}}{P_{b}}\;$$

#### 2.1.2. Orders of Cumulative Moment

Let $P_{o}^{}\left(t\right)=\sum_{i=0}^{t}\;P_{i}$ the zero-order cumulative moment of the gray-level histogram. It represents the probability of the gray level from 0 to *t.*

(6) $$P_{o}\left(t\right)=P_{o}\;$$

Making ${µ}_{o}\left(t\right)$ as the mean value of the gray level from 0 to *t* represents the first-order cumulative moment of the gray level histogram

(7) $$\mu_{o}^{}\left(t\right)=\overset{t}{\underset{i=0}{\sum }}\;\;iP_{i}\;$$

where $\mu_{T}^{}$ is the mean value of the entire image, as shown in Equation (8)

(8) $$\mu_{T}^{}=\overset{L-1}{\underset{i=0}{\sum }}\;\;iP_{i}\;$$

With Equations (1)–(8), the relation for any t can be represented in Equation (9)

(9) $$P_{o}+P_{b}=1,\;\;P_{o}\mu_{o}^{}+P_{b}\mu_{b}^{}=\mu_{T}^{}\;$$

The object class variance $\sigma_{o}^{2}\;\left(t\right)$ and the background class variance $\sigma_{b}^{2}\left(t\right)\;$can be defined using the second-order moment of the gray-level histogram, as shown in Equations (10) and (11)

(10) $$\sigma_{o}^{2}\left(t\right)=\overset{t}{\underset{i=0}{\sum }}\;\;\frac{{\left(i-\mu_{o}^{}\right)}^{2}P_{i}}{P_{o}}\;$$

(11) $$\sigma_{b}^{2}\left(t\right)=\overset{L-1}{\underset{i=t+1}{\sum }}\;\;\frac{{\left(i-\mu_{b}^{}\right)}^{2}P_{i}}{P_{b}}\;$$

#### 2.1.3. Class Variance and Total Variance

Based on the above equations, the between-class variance of object and background, the within-class variance, and the total variance are expressed as shown in Equations (12)–(14)

(12) $$\sigma_{Between-Class}^{2}=P_{o}{\left(\mu_{o}^{}-\mu_{T}^{}\right)}^{2}+P_{b}{\left(\mu_{b}^{}-\mu_{T}^{}\right)}^{2}\;$$

(13) $$\sigma_{Within-Class}^{2}=P_{o}\sigma_{o}^{2}+P_{b}\sigma_{b}^{2}\;$$

(14) $$\sigma_{Overall}^{2}=\overset{L-1}{\underset{i=0}{\sum }}\;{\left(i-\mu_{T}^{}\right)}^{2}P_{i}\;$$

Equations (12) to (14) fulfill the following relationship.

(15) $$\sigma_{Overall}^{2}\;\;\sigma_{Between-Class}^{2}\;\;\sigma_{Within-Class}^{2}\;$$

#### 2.1.4. Finding the Optimal Threshold

Based on the variance between the object and background, which is represented as $\;\sigma_{Otsu}^{2}\left(t\right)$, the maximum gray level is selected to be the optimal threshold t*, as defined in Equation (17)

(16) $$\sigma_{Otsu}^{2}=\frac{{\left[\mu_{T}^{}P_{o}\left(t\right)-\mu_{o}^{}\left(t\right)\right]}^{2}{}_{}}{P_{o}\left(t\right)\;\left[1-P_{o}\left(t\right)\right]}\;$$

(17) $$t^{*}=\arg\max\;\{\sigma_{Otsu}^{2}\;\left(t\right)\},\;0\leq t\;\leq L-1\;$$

Otsu’s method uses automatic search to find the threshold. Images with bimodal histograms can be segmented accurately, especially when the classes are represented in symmetric distribution since the Otsu method relies on the Gaussian distribution definition. However, any sudden or gradual changes between the object and background boundary can be reflected by the variance. Some medical images have more than one object that remains under the same class with overlapped gray levels in the background and may also have a right-skewed histogram, for example, some objects in MRI brain tumor images. In this case, it is difficult to select the desired gray level that separates the object from the background.

Several improvements of the Otsu method were presented in the literature. Most of the segmentation improvements were proposed for specific detection purposes. The Otsu method is improved for speediness and stabilization [11]. Otsu method was improved to study the influence of the aggregate size in asphalt concrete [12]. Otsu and Canny edge detection technique is used to determine the grain boundaries of metal [13]. The method of threshold selection combined with Otsu to calculate the volume distribution of nanoparticles in the brain parenchyma [14]. Otsu and multiple filtering techniques were used to detect the cracks in the concrete image [15]. 

In response to the stated research, some researchers have proposed an improvement for Otsu algorithms. Otsu method was improved for radiography image segmentation. Even though the threshold is located on the left bottom side of the unimodal distribution, it is only suitable for images that have a large proportion of low brightness background [10,16]. Another improvement in Otsu was presented for defect detection using the weighted variance of the object; the improvement remains sensitive to the overlapped noise in some types of images [17]. Moreover, the Otsu algorithm was proposed as a double window algorithm for mixed distribution. Nonetheless, selecting the window size is required, depending on the desired object [18]. The optimal threshold of Otsu’s algorithm and some other mean-based thresholding algorithms can completely rely on the estimated mean for their objective functions [19,20]. As a relevant work, the Otsu method was modified using Gamma and lognormal distribution, although the usage of lognormal distribution does not satisfy the lognormal definition as it only tends to replace the mean value of lognormal with the mean of Gaussian in the Otsu method [3]. The results of this modified method were also included in this research for the comparison of the original Otsu method and the proposed model. 

### 2.2. Lognormal Distribution

**Figure 1:**
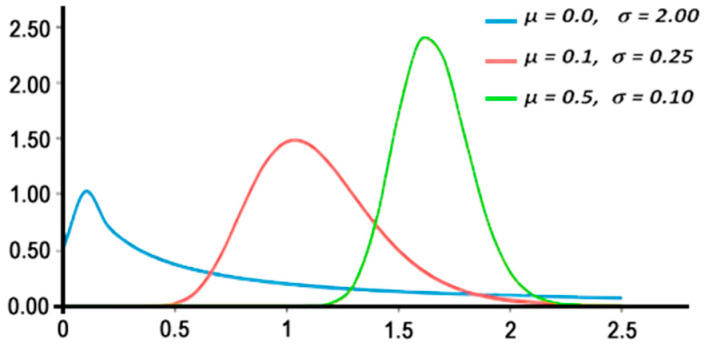
Probability density function (PDF) of three different Lognormal distributions.

The random variable whose logarithm is normally distributed has a probability distribution called the lognormal distribution. The random variable Y with a normal distribution has a lognormal distribution *X = exp (Y).* Similarly, when the variable X is log-normally distributed, the variable *Y = log (X)* is normally distributed [3]. The probability density function (PDF) of the lognormal distribution is defined in Equation (18). It is a distribution skewed to the right, where the degree of skewness increases as the standard deviation σ increases for a given mean µ. Equally, for the same σ, the skewness of the probability density function (PDF) increases as µ increases, as shown in Figure 1. Some medical images, such as ultrasound, are modeled using different distributions, including lognormal [21]. The local probability distribution function (PDF) in ultrasound images is modulated using lognormal distribution as well [22]. Lognormal is also used with region-based level sets for object separation in synthetic aperture radar (SAR) [23]. Moreover, the PDF of lognormal and the gamma distributions were used for background digital level distributions in diesel spray images [24].

(18) $$F\left(x,µ,\sigma \right)=\frac{1}{x\sigma \sqrt{2\pi }}\;e^{\frac{-1}{\;2}{\left(\frac{ln\;x-µ}{\sigma }\right)}^{2}}\;$$

where µ is the mean, σ is the standard deviation, and x is the pixel’s intensity level. As an image with histogram$\;h\left(x\right)$, it is assumed that the histogram is modeled as lognormal distributions [3,25] and can be defined in Equation (19):

(19) $$h\left(x\right)=\overset{m}{\underset{i=0}{\sum }}\;p_{i}\;f\left(x,{µ}_{i},\sigma_{i}\right),\;x>0\;$$

The $i^{th}$ mode in histogram $h\left(x\right)$ can be represented as $p_{i}f\left(x,{µ}_{i},\sigma_{i}\right)$, where $p_{i}$ is the probability of this $i^{th}$ mode, and m is the number of modes in the histogram $h\left(x\right)$. If the data in an image are assumed to be a combination of two lognormal distributions, then the mean value of the two modes in that histogram, e.g., $\mu_{o\;Log}^{}\;$and $\mu_{b\;Log}^{}$, can be estimated in Equations (20) and (21), representing the mean of the object and the background, respectively [3]

(20) $$\;\mu_{o\;Log}^{}\left(t\right)=\frac{\;\sum_{i=0}^{t}h\left(i\right).log\left(i\right)}{\sum_{i=0}^{t}h\left(i\right)}\;$$

(21) $$\mu_{b\;Log}^{}\left(t\right)=\frac{\;\sum_{i=t+1}^{L-1}h\left(i\right).log\left(i\right)}{\sum_{i=t+1}^{L-1}h\left(i\right)}\;$$

Therefore, the total mean of the entire image ${µ}_{T\;log}$ can be defined in Equation (22)

(22) $$\mu_{T\;log}^{}\left(t\right)=\frac{\;\sum_{i=0}^{L-1}h\left(i\right).log\left(i\right)}{\sum_{i=0}^{L-1}h\left(i\right)}\;$$

where $h\left(i\right)$ is the given image histogram, and L is the maximum gray level.

Concerning the previous relevant work, the original Otsu method is suitable for the symmetric histogram modes because of the usage of Gaussian distribution [4,10], as stated in Section 2.1. Otsu method was improved in [3,25] using only the mean of lognormal inside the original Otsu’s method that follows the Gaussian definition, which is not true when thresholding images with right-skewed intensities. In this work, we have corrected the stated issue by developing a formula of between-class variance based on lognormal distribution. The proposed formula is more general than using Gaussian distribution when applying images with right-skewed histograms for segmentation. The proposed model aims to evolve the thresholding function based on the mean and the variance of the lognormal. Therefore, it improves thresholding efficacy for images that have right-skewed histograms. The strength of the proposed model against related methods is represented in the impact of the mean and the variance using lognormal distribution, as shown in Table 1. 

## 3. Materials and Methods

As stated in the Introduction and the related work, it is a difficult task to analyze the shape of a specific object in complex scenes of an image, especially when using traditional segmentation techniques. The Otsu method was improved several times in literature [3,10,16,17,18]. However, Otsu is more suitable for images with symmetric distribution because it relies on the definition of Gaussian distribution. This paper aims to develop a formula for between-class variance and boost the optimality of the threshold value for the image segmentation process. The proposed formula is derived based on the definition of the lognormal distribution, in which the mean and the variance are based on the lognormal definition. The proposed model tends to avoid the usage of the Gaussian distribution in the between-class variance. Therefore, the proposed between-class variance using lognormal distribution has been developed as a formula on which the optimal threshold maximizes. The proposed model aims to improve the segmentation accuracy of specific cases when images have a right-skewed histogram.

### 3.1. Materials

Images with a right-skewed histogram have been used for segmentation to conduct the objective evaluation and the efficiency comparison of the proposed model with the relevant works in this regard. The used datasets represent an asymmetric distribution of gray levels. In this study, we applied 50 simulated images and 100 MRI brain tumor images for segmentation in parallel with the proposed model and relevant methods. The sizes of the used images are 256 × 256 pixels. The MRI brain tumor images are part of the public archives from the database BRATS2012, 2015, and the Harvard Medical School website [7]. They were applied for segmentation using Minimum Cross Entropy Thresholding (MCET) with heterogeneous mean filters [20]. We will not compare the results with that study because they use different notions. Images are selected for segmentation in this study based on their right-skewed histogram.

### 3.2. Proposed Between-Class Variance

According to the definition of the between-class variance in Section 2.1, the usage of Gaussian distribution with the Otsu method is more suitable with the symmetric distribution but not with the asymmetric or right-skewed distribution, where the process of mean calculation lies under a classical mean definition, and the variance lies under the definition Gaussian distribution. Furthermore, according to the definition of lognormal distribution in Section 2.2, the mean and the variance are more suitable for the cases where images have right-skewed histograms. Based on the definition of the lognormal distribution, the mean value of two modes in a given image can be estimated using Equations (20) and (21). Using this right-skewed distribution is more general than the Gaussian distribution in terms of dealing with right-skewed histograms. Having the definition of the between-class variance in the original Otsu method, our developed formula $\sigma_{Lognormal}^{2}\left(t\right)$ aims to use the mean and the variance of the lognormal distribution, as defined in Equation (23)

(23) $$\sigma_{Lognormal}^{2}\left(t\right)=P_{o}\left[log\;(\mu_{o\;Log}^{}\left(t\right)\right)-\mu_{T\;log}^{}\left(t\right){]}^{2}+P_{b}\left[log\;(\mu_{b\;Log}^{}\left(t\right)\right)-\mu_{T\;log}^{}\left(t\right){]}^{2}\;$$

where $\mu_{o\;log}^{}\left(t\right)$, $\mu_{b\;log}^{}\left(t\right)$, and $\mu_{T\;log}^{}\left(t\right)$ are the mean values based on the definition of the lognormal distribution, as stated in Equations (20)–(22), respectively; this is when the data in the histogram are modeled as a lognormal distribution as stated in Equation (19). 

The variance between object and background in the proposed between-class variance is defined as$\;\sigma_{Lognormal}^{2}\left(t\right)$ in Equation (23). The maximum gray level t* is selected using the sequential search function defined in Equation (24) as the desired value corresponding to maximum variance, representing the optimal threshold in the proposed model.

(24) $$t^{*}=\arg\max\;\{\sigma_{Lognormal}^{2}\;\left(t\right)\},\;0\leq t\;\leq L-1\;$$

**Figure 2:**
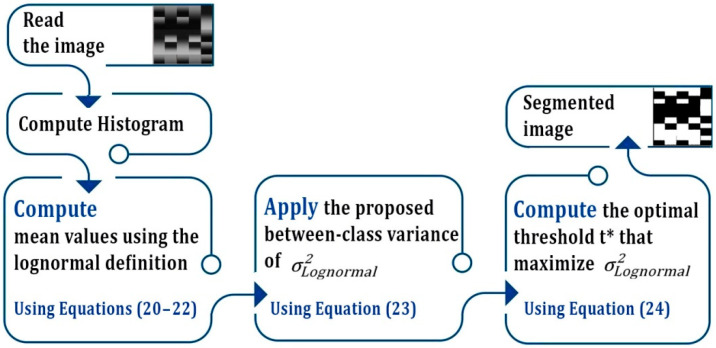
Workflow schema of the proposed segmentation model.

Figure 2 shows the workflow schema of the proposed between-class variance.

### 3.3. Proposed Algorithm

The proposed model has been implemented with MATLAB R2019a 64-bit and MATLAB parallel computing toolbox. Intel Core i5 quad-core, 3.8 GHz, and 8 GB RAM. The proposed model is applied to an image with N pixels and L intensities. The optimal threshold t* is computed by maximizing the $\sigma_{Lognormal}^{2}\left(t\right)$). Therefore, the time complexity of the proposed algorithm is the maximum of (O(N), O[L∗L])To improve time performance, the algorithm can use a fast recursive dynamic programming algorithm, especially when applying large datasets for segmentation. The overall computational processes can perform better than sequential processing, as shown in Table 2. The pseudocode for the proposed algorithm is Algorithm 1.
**Algorithm 1.** Parallel Processing1. Input image f(𝑥, 𝑦) 2. Compute the histogram h(i), i = 0, …., 255 for f(𝑥, 𝑦) 3. Parfor t = 0: 255 do 4. Compute $\mu_{o\;Log}$(t) using Equation (20) 5. Compute $\mu_{b\;Log}$(t) using Equation (21) 6. Compute $\mu_{T\;Log}$(t) using Equation (22) 7. Compute $\sigma_{Lognormal}^{2}\left(t\right)$ using Equation (23). 8. Compute the original and the modified $\sigma_{Otsu}^{2}\left(t\right)$ based on their distributions. 9. Find the optimal t* which maximizes each of $\sigma_{Lognormal}^{2}\left(t\right)$ and the relevant $\sigma_{Otsu}^{2}\left(t\right)$ 10. End for. 11. Compute the average sum of the performance measure for each t*. 12. Find the best t* which maximizes the performance measure. 13. Return the best t* 14. Output image g(𝑥, 𝑦).

## 4. Performance Evaluation

### 4.1. Unsupervised Evaluation

Evaluating segmentation results can be achieved without any a priori image data. The evaluation that relies on the statistical approach between the segmented and the original images is the unsupervised evaluation, such as uniformity and region contrast [26,27].

#### 4.1.1. Image Uniformity (IU)

This evaluation helps to measure the quality of the thresholding method. Region uniformity can be computed based on the variance in a given image. It was proposed by Levine et al. [26], as shown in Equation (25)

(25) $$IU\left(t\right)=1-\frac{\sigma_{1}^{2}\left(t\right)-\sigma_{2}^{2}\left(t\right)}{Z}$$

where *Z* is calculated as shown in Equation (26), and $\sigma_{1}^{2}\left(t\right)\;\mathrm{and}\;\sigma_{2}^{2}\left(t\right)$ are the variances of$\;mode_{a}$ and $mode_{b}$, respectively,

(26) $$Z=\frac{{\left(I_{max}-I_{min}\right)}^{2}}{2}$$

where $I_{max}$ and $I_{min}$ are the maximum and the minimum intensities.

#### 4.1.2. Region Contrast (RC)

This evaluation finds the adjacent regions and checks the high contrast; thus, it measures the quality of segmented results, as shown in Equation (27)

(27) $$\;RC\left(t\right)=\frac{\left|\mu_{1}^{}\left(t\right)-\mu_{2}^{}\left(t\right)\right|}{\mu_{1}^{}\left(t\right)-\mu_{2}^{}\left(t\right)}$$

where $\mu_{1}\left(t\right)$ and $\mu_{2}\left(t\right)$ are the mean values of the regions 1 and 2 in a given image, respectively. The value of RC(t) ranges between 0 and 1 such that 0 indicates pore segmentation and 1 indicates accurate segmentation.

### 4.2. Supervised Evaluation

**Figure 3:**
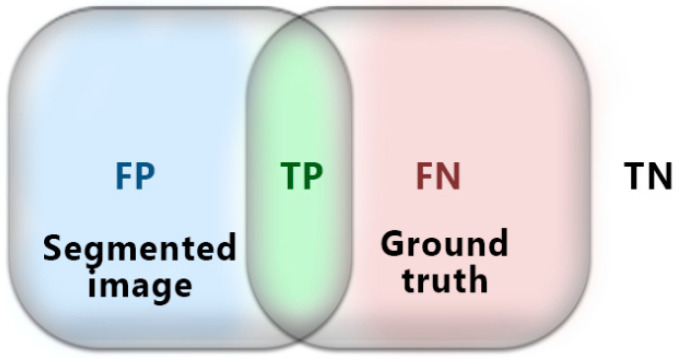
TN refers to pixels that have not been segmented, FN refers to pixels that should belong to the segmented image, TP refers to the joint segmented pixels, and FP refers to pixels that should not have been segmented.

This evaluation depends on the pixels matching approach between the segmented results and their reference or the ground truth. It is a widely used approach in the literature and is considered a powerful measurement for segmentation quality [28,29,30,31]. The aim of using this evaluation is to maximize the true positivity (TP) of pixels in the segmentation result based on the terms shown in Figure 3. 

The Jaccard index in Equation (28) helps to evaluate the intersection percent of F-scores in Equation (29), which is to evaluate the true positive pixels and their probability, where precision represents the TP/TP + FP, and recall represents the TP/TP + FN. Precision helps to measure the detected true pixels, and recall helps to measure the true positivity and the matching rate and whether the segmented pixel belongs to the ground truth. Segmentation accuracy refers to the accurate match between the pixels in the segmented image and its reference to the ground truths, as shown in Equation (30)

(28) $$\;Jaccard_{index}=\frac{TP}{TP+FP+FN}\;$$

(29) $$\;F_{Score}=2*\frac{Precision\;Recall}{Precision+Recall}$$

(30) $$\;Accuracy=\frac{TP+TN}{FN+FP+TP+TN}$$

### 4.3. Modeling the Accurate Segmentation

The lognormal distribution has been used to form the between-class variance of the proposed model so that the threshold value reaches the maximum, and this function is compared with the between-class variance of the original Otsu method using Gaussian distribution. When the value t is maximized, it indicates the final threshold t* for the given image, and the accurate segmentation aims to maximize the evaluation scores; we propose using the parallel processing evaluation to reduce time complexity in each segmentation case, as follows:

(31) $$\mathrm{Maximize}\;\mathrm{Evaluation}\left(\mathrm{Unsupervised},\;\mathrm{Supervised}\right)$$

where unsupervised (IU(t), RC(t)), and supervised evaluations ($Jaccard_{index},\;F_{Score},\;Acuuracy)\;$∈ [0, 1].

## 5. Results and Discussion

**Figure 4:**
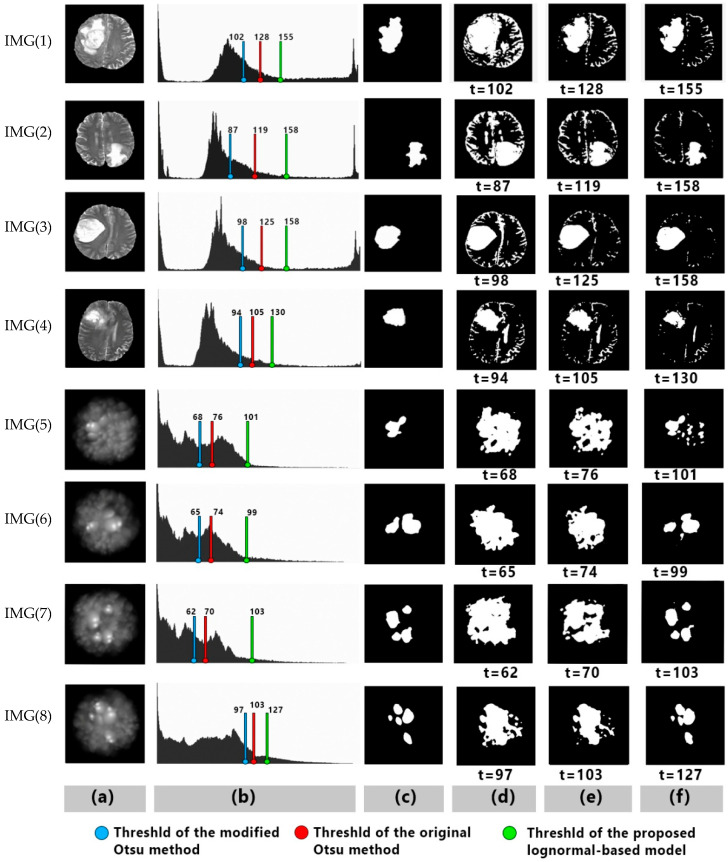
Selected samples, IMGs (1), (2), (3), and (4) are MRI brain tumor images, IMGs (5), (6), (7), and (8) are simulated images; (**a**) are original images, (**b**) corresponding histograms, (**c**) ground truths, (**d**) segmentation results using the modified Otsu method [3], (**e**) segmentation results using the original Otsu, and (**f**) segmentation results using the proposed lognormal-based model.

Several tests were performed for segmentations; they were evaluated to attain the segmentation efficiency of the proposed model. To show the significance of the proposed model against the original and the relevant methods, two types of measurement were used, unsupervised and supervised evaluations, to obtain maximum scores for each segmented image. Figure 4 shows selected samples of segmented images that have a right-skewed histogram. These samples reflect experimental results subjectively when comparing results from the proposed model with the original and modified Otsu methods, as shown in columns (c) and (f) in Figure 4. Nonetheless, it can be noticed that the objective results appeared in the evaluation scores, as shown in Table 3. 

The overall experimental results showed appreciable objective results. From the point of view of the mean-based thresholding, the type of the distribution does matter; thus, finding the optimal threshold in images with right-skewed intensity levels seems to be a challenging task when using the Gaussian distribution with the Otsu method. It is noticeable that both types of images used in this research have histograms skewed right, and there was a wide gap in obtaining the optimal threshold, as shown in Figure 4b. 

In the original and the modified Otsu methods, the thresholds were shifted to the left, including pixels from the fuzzy backgrounds. It can be noticed that the gap was filled when using the proposed model, where the threshold value acts to exclude most of the unwanted area between the object and the background. Simulated images were used in this work to focus on the goal of segmenting images with a right-skewed histogram, as it has wide skewness covering the range of the intensity levels. 

In most cases, the supervised evaluation shows a slight increase in scores against the unsupervised evaluation, as shown in Table 3. The slight difference depends on the process used in each measurement. The unsupervised evaluation relies on the statistical approach between the output image and the structure of the original image. In contrast, the supervised evaluation relies on the matching approach between pixels in the output image and the ground truth, e.g., the recorded scores of the unsupervised evaluation of IMG (1) using the proposed model are 0.86912 IU and 0.88931 RC, while the recorded scores of the supervised evaluation are 0.89927 Jaccard index, 0.90158 F-scores, and 0.91067 segmentation accuracy. 

The overall evaluation was computed by averaging each evaluation, as shown in Table 4. The three methods and their corresponding evaluations were recorded in four patterns. Two evaluations for simulated images and two for MRI brain tumor images, referring to the unsupervised and the supervised evaluations. As can be noticed from the results, the proposed model yield better thresholding accuracy and promising image segmentation outputs. 

The generality of the performance evaluation in Table 4 indicates that simulated image segmentation showed a 2.19% accuracy increase rate with the unsupervised evaluation and 2.30% with the supervised evaluation compared with the original method. In MRI brain tumor image segmentation, the proposed model showed a 4.00% accuracy rate increase with the unsupervised evaluation and 2.13% with the supervised evaluation. Overall, the between-class variance using lognormal was able to record better evaluation scores over the other methods for such kinds of images. This effective increase rate reflects the subjective and objective goals of accurate segmentation for images with a right-skewed histogram. 

## 6. Conclusions and Future Work

This paper presents an improvement of between-class variance based on the mean and variance of the lognormal distribution. The proposed model aims to find the optimal threshold in right-skewed histograms for accurate image segmentation. Simulated images and MRI brain tumor data sets have been applied for segmentation and evaluation. The results of the proposed model were examined using the supervised and unsupervised evaluation in comparison with the original and related methods. Based on the used evaluation, the proposed model showed an accuracy increase rate of 2.24% when applying MRI brain tumor images and 3.00% when applying simulated images for segmentation against relevant methods. This paper contributes to improving the segmentation results of asymmetric and right-skewed distribution for different images.

In future work, the proposed model can be examined by applying a large dataset of extended types of medical and optical images for multimodal thresholding and extending the contribution to a wide domain of applications for image segmentation.

## Figures and Tables

**Table 1:** Effectiveness of the proposed method compared with the related methods.

Between-Class Variance	Parameters Values	Effectiveness
The Original Otsu	Mean and variance are based on the definition of Gaussian distribution	Suitable for images with symmetric distribution but limited for asymmetric distributions
The Modified Otsu [3]	The original Otsu formula using only the mean value of the lognormal distribution	Almost have the same efficiency as the original method with improvements for certain types of images
The Proposed Model	Mean and variance are based on the definition of Lognormal distribution	Boosted efficacy for images with right-skewed intensity distributions but not suitable for left-skewed distribution.

**Table 2:** Time performance of the proposed algorithm when implemented using parallel processing.

Segmented Images		No. of Images	Sequential(s)	Parallel(s)	Speedup Gain
1	MRI Brain Tumor	100	407.904	267.552	34.4%
3	Simulated Images	50	301.710	203.391	32.5%

**Table 3:** Evaluation scores of images in Figure 4: (A) refers to supervised evaluation, and (B) refers to unsupervised evaluation.

	Modified Otsu [3]		Original Otsu		The Proposed Model	
	A	B	A	B	A	B
IMG(1)	0.87977	0.84472	0.88948	0.85621	0.90387	0.87921
IMG(2)	0.86998	0.84380	0.87650	0.85757	0.91105	0.87987
IMG(3)	0.85892	0.83877	0.87380	0.85591	0.90744	0.88760
IMG(4)	0.86390	0.82988	0.88157	0.85608	0.90094	0.87794
IMG(5)	0.84898	0.82790	0.87599	0.86189	0.91079	0.89093
IMG(6)	0.83907	0.82319	0.85280	0.86839	0.91969	0.88957
IMG(7)	0.82995	0.81758	0.85720	0.86898	0.92781	0.89116
IMG(8)	0.83614	0.82947	0.84997	0.85911	0.91570	0.88931

**Table 4:** Evaluation of performance measures for the original Otsu method, the modified Otsu method [3], and the proposed model.

	Original Otsu	Modified Otsu [3]		The Proposed Model	
Images/Evaluation	Average Metrics	Average Metrics	Accuracy Increase rate	Average Metrics	Accuracy Increase rate
MRI Brain Tumor/Unsupervised	0.87028	0.8681	−0.25%	0.88931	2.19%
MRI Brain Tumor/Supervised	0.88177	0.87903	−0.31%	0.90208	2.30%
Simulated Images/Unsupervised	0.87593	0.87470	−0.14%	0.91097	4.00%
Simulated Images/Evaluation	0.89298	0.89109	−0.21%	0.91208	2.13%

## Data Availability

Data presented in this study are available on request.
